# Structure of [60]fullerene with a mobile lithium cation inside

**DOI:** 10.1098/rsos.180337

**Published:** 2018-07-18

**Authors:** Shinobu Aoyagi, Kazuhira Miwa, Hiroshi Ueno, Hiroshi Okada, Yutaka Matsuo, Ken Kokubo

**Affiliations:** 1 Department of Information and Basic Science, Nagoya City University, Nagoya 467-8501, Japan; 2 School of Chemistry, Northeast Normal University, 5268 Renmin Street, Changchun, Jilin 130024, People's Republic of China; 3 Department of Mechanical Engineering, School of Engineering, The University of Tokyo, 7-3-1 Hongo, Bunkyo-ku, Tokyo 113-8656, Japan; 4 Hefei National Laboratory for Physical Sciences at the Microscale, University of Science and Technology of China, 96 Jinzhai Road, Hefei, Anhui 230026, People's Republic of China; 5 Division of Applied Chemistry, Graduate School of Engineering, Osaka University, Suita, Osaka 565-0871, Japan

**Keywords:** fullerene, endohedral metallofullerene, Li^+^@C_60_, X-ray crystal structure analysis, synchrotron radiation

## Abstract

The structure of crystalline [60]fullerene with a lithium cation inside (Li^+^@C_60_) was determined by synchrotron radiation X-ray diffraction measurements to understand the electrostatic and thermal properties of the encapsulated Li^+^ cation. Although the C_60_ cages show severe orientation disorder in [Li^+^@C_60_](TFPB^−^)·C_4_H_10_O and [Li^+^@C_60_](TFSI^−^)·CH_2_Cl_2_, the Li^+^ cations are rather ordered at specific positions by electrostatic interactions with coordinated anions outside the C_60_ cage. The Li^+^@C_60_ molecules in [Li^+^@C_60_](ClO_4_^−^) with a rock-salt-type cubic structure are fully disordered with almost uniform spherical shell charge densities even at 100 K by octahedral coordination of ClO_4_^−^ tetrahedra and show no orientation ordering, unlike [Li^+^@C_60_](PF_6_^−^) and pristine C_60_. Single-bonded (Li^+^@C_60_^−^)^2^ dimers in [Li^+^@C_60_^−^](NiOEP)⋅CH_2_Cl_2_ are thermally stable even at 400 K and form Li^+^–C bonds which are shorter than Li^+^–C bonds in [Li^+^@C_60_](PF_6_^−^) and suppress the rotational motion of the Li^+^ cations.

## 1. Introduction

Hollow spherical carbon molecules called fullerenes or buckyballs with a molecular formula of C_2*n*_ (*n* ≥ 30) can contain various atoms and molecules [1,2]. Endohedral fullerenes containing atoms and molecules with magnetic and electric moments have the potential to be used for molecular devices such as quantum bits [3,4], magnetic resonance imaging agents [5,6] and molecular switches [7,8]. [60]Fullerene C_60_ with a soccer-ball shape of 1 nm diameter is the most abundant fullerene in soot obtained by arc discharge of graphite electrodes and is valuable in applications such as solar cells [9,10]. Endohedral C_60_ with a lithium cation inside (Li^+^@C_60_) is efficiently obtained by lithium plasma bombardment with C_60_ vapour onto a target plate and is commercially available from Idea International Co., Ltd. [11–13]. It has been reported that Li^+^@C_60_ shows outstanding electron-accepting properties for applications such as photoelectrochemical solar cells [14–16] and non-volatile organic transistor-based memories [17].

Li^+^@C_60_ forms crystalline salts with anions such as SbCl_6_^−^ and PF_6_^−^ [11,18]. The PF_6_^−^ salt, [Li^+^@C_60_](PF_6_^−^), has a rock-salt-type cubic structure and shows a fast response to an alternating electric field by free rotational motion of the Li^+^ cation on a shell with a radius of 1.5 Å inside the C_60_ cage [18–20]. The free rotational motion of the Li^+^ cation is suppressed so that it is localized at two off-centre equivalent positions on the threefold inversion axis of the cubic crystal below 100 K. The simultaneous occupation of the two positions at low temperature is attributed to a quantum tunnelling of the Li^+^ cation between the two positions with an interval of 2.7 Å. The tunnelling motion is also hindered below *T*_C_ = 24 K with an abrupt decrease in the dielectric permittivity by antiferroelectric interactions among local electric dipole moments formed between the Li^+^ cations inside and the PF_6_^−^ anions outside the C_60_ cages. The tunnelling motion and intermolecular interaction of the Li^+^ cation suggest that Li^+^@C_60_ has the potential to be used as a quantum bit in quantum computers using electric dipole moments.

The position and motion of a Li^+^ cation inside C_60_ are significantly affected by the coordinated anions and molecules outside the C_60_. For instance, the Li^+^ cation in the SbCl_6_^−^ salt is localized at two adjoining off-centre positions at 370 K by asymmetric coordination of SbCl_6_^−^ anions around an Li^+^@C_60_ molecule [11]. Such anion exchange effects should be investigated in more depth to understand the electrostatic responses of the encapsulated Li^+^ cations. Crystals of [Li^+^@C_60_](PF_6_^−^), [Li^+^@C_60_](SbCl_6_^−^) and [Li^+^@C_60_](TFPB^−^) (TFPB, tetrakis{3,5-bis(trifluoromethyl)phenyl}borate) were previously obtained and structurally characterized [11,18,21]. Further structural study of [Li^+^@C_60_](TFPB^−^) is required, because the position and motion of the Li^+^ cation have not been revealed in the previous structural analysis [21]. The anions of the previously obtained crystals are all non-polar. Li^+^@C_60_ crystals combined with polar anions such as TFSI^−^ (TFSI, bis(trifluoromethylsulfonyl)imide) should also be obtained and structurally characterized. PF_6_^−^ and SbCl_6_^−^ are small octahedral anions, whereas TFPB^−^ is a large tetrahedral anion. It is worthwhile obtaining and investigating Li^+^@C_60_ crystals combined with small tetrahedral anions such as ClO_4_^−^. A crystal of dimerized Li^+^@C_60_^−^ without anions was also obtained by electrochemical reduction [22]. The crystal is different from other dimerized endohedral metallofullerene crystals that consist of negatively charged endohedral metallofullerenes and positively charged donor molecules [23,24]. The Li^+^ cation in the dimerized Li^+^@C_60_^−^ is localized near the carbon atom that is nearest to the carbon atom forming an inter-fullerene single C–C bond at 100 K. Temperature dependence of the position and motion of the localized Li^+^ cation in the dimerized Li^+^@C_60_^−^ would provide us with essential information about the thermal stability of the (Li^+^@C_60_^−^)^2^ dimer and the Li^+^–C bond.

To understand the electrostatic and thermal properties of the mobile Li^+^ cation inside C_60_, we investigated the effects of the coordination structure and temperature on the position and motion of the encapsulated Li^+^ cations in Li^+^@C_60_ crystals by synchrotron radiation (SR) X-ray structure analysis in this study.

## 2. Material and methods

We performed the X-ray crystal structure analyses of [Li^+^@C_60_](TFPB^−^), [Li^+^@C_60_](TFSI^−^), [Li^+^@C_60_](ClO_4_^−^) and [Li^+^@C_60_^−^](NiOEP) (OEP, octaethylpolphyrine). The TFPB^−^, TFSI^−^ and ClO_4_^−^ salts were obtained by anion exchange of [Li^+^@C_60_](PF_6_^−^) [21]. Single crystals of the TFPB^−^ and TFSI^−^ salts and powder crystals of the ClO_4_^−^ salt were obtained by vapour diffusion from solutions. The crystal structure of [Li^+^@C_60_](TFPB^−^) without solvent molecules at 123 K has already been reported [21]. A single crystal of [Li^+^@C_60_](TFPB^−^) containing diethyl ether solvent molecules was obtained in this study. Single crystals of [Li^+^@C_60_^−^](NiOEP) were obtained by electrochemical reduction in [Li^+^@C_60_](TFSI^−^) solution in the presence of NiOEP [22]. The crystal structure of [Li^+^@C_60_^−^](NiOEP) containing dichloromethane solvent molecules at 100 and 250 K has already been reported [22]. We report the crystal structure of [Li^+^@C_60_^−^](NiOEP) at 400 K and discuss the temperature dependence of the crystal structure in this study. The SR X-ray diffraction (XRD) measurements were performed at beamline BL02B1 of the SPring-8 large SR facility [25]. The crystal structures were determined by using *SIR* [26], *SHELX* [27] and *SP* [28]. The experimental conditions and crystallographic data are summarized in table 1 and crystallographic information files (CIFs) as the electronic supplementary material. The CIF deposition numbers at the Cambridge Crystallographic Data Centre (CCDC) are 1826722 for [Li^+^@C_60_](TFPB^−^), 1826723 for [Li^+^@C_60_](TFSI^−^) and 1826724 for [Li^+^@C_60_^−^](NiOEP).

**Table 1.** Experimental conditions and crystallographic data. The reliable factors based on absolute structure factors (*R*) and the weighted reliable factors based on squared structure factors (*R*_w_) are given for the single-crystal structure refinements of [Li^+^@C_60_](TFPB^−^), [Li^+^@C_60_](TFSI^−^) and [Li^+^@C_60_^−^](NiOEP). The weighted profile reliable factor (*R*_wp_) and the reliable factor based on Bragg intensities (*R*_I_) are given for the Rietveld refinement of [Li^+^@C_60_](ClO_4_^−^).

	[Li^+^@C_60_](TFPB^−^)	[Li^+^@C_60_](TFSI^−^)	[Li^+^@C_60_](ClO_4_^−^)	[Li^+^@C_60_^−^](NiOEP)
formula	LiC_60_·C_32_H_12_BF_24_·	LiC_60_·C_2_F_6_NO_4_S_2_·	LiC_60_·O_4_Cl	LiC_60_·NiC_36_N_4_H_44_·
	C_4_H_10_O	CH_2_Cl_2_		CH_2_Cl_2_
formula weight	1664.88	1092.62	827.03	1403.93
crystal size (mm)	0.25 × 0.12 × 0.11	0.30 × 0.08 × 0.04	powder	0.25 × 0.03 × 0.03
temperature (K)	260	150	30–450	400
X-ray wavelength (Å)	0.41324	0.70220	0.64898	0.41400
crystal system	monoclinic	orthorhombic	cubic	monoclinic
space group	*P*2_1_/*c*	*Pnma*	$Fm\bar{3}m$	*C*2/*m*
unit cell parameters	*a* = 17.1122(9) Å	*a* = 18.963(5) Å	*a* = 14.133(1) Å	*a* = 24.6791(13) Å
	*b* = 20.3829(12) Å	*b* = 13.892(3) Å	*V* = 2822.9(6) Å^3^	*b* = 14.9697(6) Å
	*c* = 19.8912(18) Å	*c* = 13.964(3) Å	(100 K)	*c* = 17.7061(15) Å
	*β* = 105.157(5)°	*V* = 3678.5(15) Å^3^		*β* = 109.171(5)°
	*V* = 6696.6(8) Å^3^			*V* = 6178.5(7) Å^3^
*Z*	4	4	4	4
no. of independent reflections	13 115 (*d* > 0.80 Å)	3684 (*d* > 0.78 Å)	162 (*d* > 0.85 Å)	8779 (*d* > 0.70 Å)
			(1434 data points)	
no. of parameters	1459	433	55	657
reliable factors	*R* = 0.125 (|*F*| > 4*σ*)	*R* = 0.098 (|*F*| > 4*σ*)	*R*_wp_ = 0.036 (100 K)	*R* = 0.067 (|*F*| > 4*σ*)
	*R*_w_ = 0.363 (|*F*| > 4*σ*)	*R*_w_ = 0.312 (|*F*| > 4*σ*)	*R*_I_ = 0.124 (100 K)	*R*_w_ = 0.200 (|*F*| > 4*σ*)

## 3. Results and discussion

### 3.1. [Li^+^@C_60_](TFPB^−^)·C_4_H_10_O

**Figure 1.**
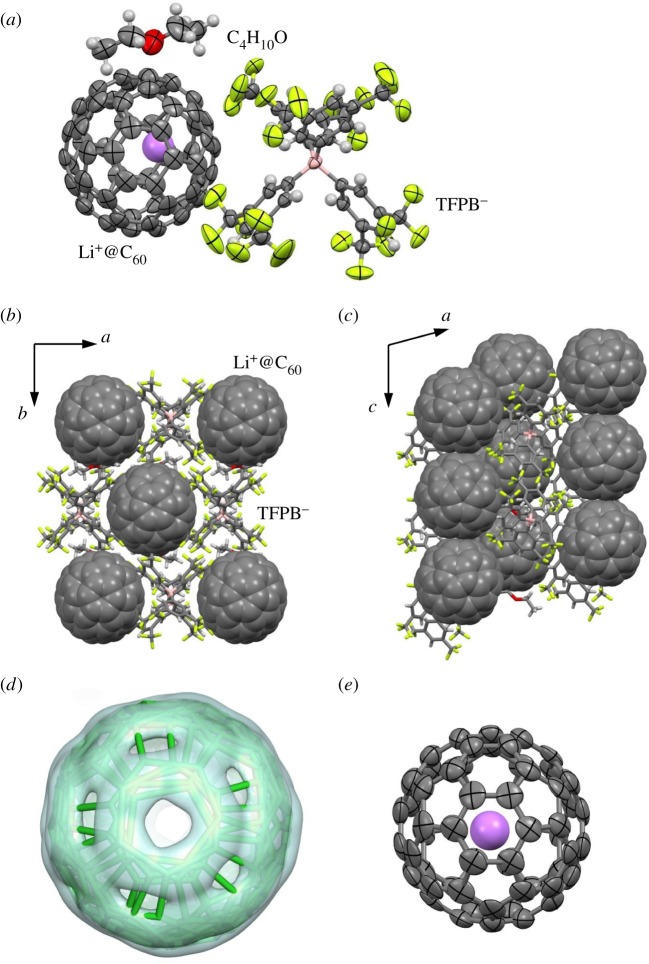
Crystal structure of [Li^+^@C_60_](TFPB^−^)·C_4_H_10_O at 260 K. (*a*) Structure with thermal ellipsoids at the 50% probability level viewed along the *c*-axis. Hydrogen atoms are drawn as small spheres. Disordered structures are omitted. (*b*) Molecular arrangement viewed along the *c*-axis. (*c*) Molecular arrangement viewed along the *b*-axis. (*d*) Disordered structure of the C_60_ cage with the electron charge-density surface at 1.5 *e*/Å^3^ obtained by the maximum entropy method. A pentagon of the major orientation and four hexagons of the minor orientations are overlapping in the structure. (*e*) Structure of Li^+^@C_60_ with thermal ellipsoids at the 50% probability level viewed perpendicular to a hexagon near the violet encapsulated Li^+^ cation.

Figure 1 shows the crystal structure of [Li^+^@C_60_](TFPB^−^) containing diethyl ether (C_4_H_10_O) solvent molecules at 260 K. The crystal shows a structural phase transition with a non-merohedral twinning around 250 K. The crystal structure above the phase transition temperature was determined in this study. The monoclinic lattice constants (*a* = 17.11 Å, *b* = 20.38 Å, *c* = 19.89 Å, *β* = 105.16°) and molecular arrangement are rather different from those of a solvent-free crystal (*a* = 13.40 Å, *b* = 26.03 Å, *c* = 17.03 Å, *β* = 90.52°) reported in [21]. The volume of the unit cell containing four [Li^+^@C_60_](TFPB^−^) ion pairs for the solvent-containing crystal (6697 Å^3^) is larger than that for the solvent-free crystal (5939 Å^3^). As a result, the coordination structures around the Li^+^@C_60_ are different between the solvent-free and solvent-containing crystals. The C_60_ centres are in the general positions of monoclinic structures in both crystals. The Li^+^@C_60_ molecule in the solvent-free crystal is coordinated by six TFPB^−^ anions with an octahedral arrangement. The distance from the C_60_ centre to the nearest central boron atom of TFPB^−^ is 8.1 Å. The distance from the C_60_ centre to the nearest C_60_ centre is 11.4 Å. The crystal structure of the solvent-containing [Li^+^@C_60_](TFPB^−^) consists of one-dimensional Li^+^@C_60_ arrays and TFPB^−^ arrays along the *c*-axis, as shown in figure 1*b*,*c*. The distance from the C_60_ centres to the nearest central boron atom of TFPB^−^ is 8.7 Å. The distance from the C_60_ centre to the nearest two C_60_ centres along the *c*-axis is 10.0 Å. Therefore, Li^+^@C_60_ to TFPB^−^ distances are increased and Li^+^@C_60_ to Li^+^@C_60_ distances are decreased by intercalation of C_4_H_10_O solvent molecules.

The differences in coordination structure around Li^+^@C_60_ between the solvent-free and solvent-containing crystals affect the rotational motion of the C_60_ cages. Fullerene molecules often show rotational motion and orientation disorder in crystals. For instance, C_60_ cages in [Li^+^@C_60_](PF_6_^−^) and pristine C_60_ crystals show free rotational motion at high temperature [18,29]. The free rotational motions are stopped at low temperature by phase transitions at 370 K in [Li^+^@C_60_](PF_6_^−^) and 260 K in pristine C_60_. Although the C_60_ orientation is perfectly ordered in the low temperature [Li^+^@C_60_](PF_6_^−^) [18], orientation disorder with two molecular orientations remains in the low-temperature pristine C_60_ [30,31]. This suggests that rotational motion of the C_60_ cages is hindered by electrostatic interactions between the cationic Li^+^@C_60_ and coordinated anions. C_60_ cages in the reported crystal structure of the solvent-free [Li^+^@C_60_](TFPB^−^) show no orientation disorder [21]. The electrostatic interactions between Li^+^@C_60_ and TFPB^−^ should be weakened by the increase in Li^+^@C_60_ to TFPB^−^ distances by intercalation of C_4_H_10_O solvent molecules. As a result, C_60_ cages in the solvent-containing [Li^+^@C_60_](TFPB^−^) crystal show a severe orientation disorder with five molecular orientations, as shown in figure 1*d*. The site occupancies for the five C_60_ orientations are 0.372(5), 0.253(4), 0.155(4), 0.132(3) and 0.124(4).

A weak electron charge-density peak for an Li^+^ cation was observed inside the disordered C_60_ cage of the solvent-containing [Li^+^@C_60_](TFPB^−^) at 260 K. The Li^+^ position at (*x*, *y*, *z*) = (0.235, 0.758, 0.151) is close to the gravity centre at (0.228, 0.741, 0.147) for the central boron atoms of the six TFPB^−^ anions adjacent to the Li^+^@C_60_ with an interval of 0.13 Å, and, hence, the Li^+^ position is electrostatically consistent with the anion arrangement. The Li^+^ position is beneath the centre of a hexagon of the C_60_ with the major orientation, as shown in figure 1*e*. The average Li^+^–C distance is 2.35(10) Å, which agrees with the value for [Li^+^@C_60_](PF_6_^−^) at low temperature [18,19]. Assuming that the site occupancy for the Li^+^ position is the same as that of the major C_60_ orientation (0.372(5)), the isotropic atomic displacement parameter of the Li^+^ is refined to 0.24(3) Å^2^, and the remaining fraction of the Li^+^ cation would occupy other positions inside the C_60_ by a positional disorder. The Li^+^ position in the solvent-free [Li^+^@C_60_](TFPB^−^) has not been determined even at 123 K [21]. The Li^+^ cation of cubic [Li@C_60_](PF_6_^−^) is freely rotating on a shell with a radius of 1.5 Å inside the C_60_ cage above 100 K [18–20]. Therefore, the Li^+^ cation is partially ordered in the solvent-containing [Li^+^@C_60_](TFPB^−^) by the asymmetric coordination of TFPB^−^ anions and C_4_H_10_O molecules, as shown in figure 1*b,c*.

### 3.2. [Li^+^@C_60_](TFSI^−^)·CH_2_Cl_2_

**Figure 2.**
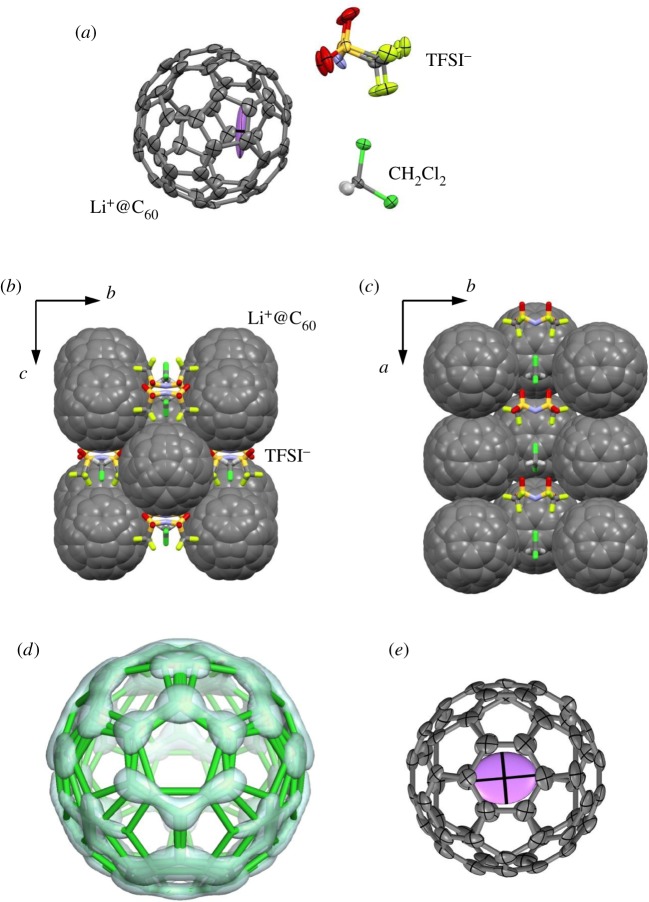
Crystal structure of [Li^+^@C_60_](TFSI^−^)·CH_2_Cl_2_ at the 150 K. (*a*) Structure with thermal ellipsoids at the 50% probability level viewed along the *b*-axis. Hydrogen atoms are drawn as small spheres. Disordered structures are omitted. (*b*) Molecular arrangement viewed along the *a*-axis. (*c*) Molecular arrangement viewed along the *c*-axis. (*d*) Disordered structure of the C_60_ cage with the electron charge-density surface at 1.7 *e*/Å^3^ obtained by the maximum entropy method. Two hexagons of the major orientations and a pentagon of the minor orientation are overlapping in the structure. (*e*) Structure of Li^+^@C_60_ with thermal ellipsoids at the 50% probability level viewed perpendicular to a hexagon near the violet encapsulated Li^+^ cation.

Figure 2 shows the crystal structure of [Li^+^@C_60_](TFSI^−^) containing dichloromethane (CH_2_Cl_2_) solvent molecules at 150 K. The crystal shows a structural phase transition with a non-merohedral twinning around 130 K. The crystal structure above the phase transition temperature was determined in this study. The TFSI^−^ anion and CH_2_Cl_2_ molecule are polar molecules, while the TFPB^−^, ClO_4_^−^, SbCl_6_^−^ and PF_6_^−^ anions are non-polar molecules. Permanent electric dipole moments of such polar molecules should electrostatically interact with the Li^+^ cations inside the C_60_ cages in crystals. However, the electric dipole moments of TFSI^−^ and CH_2_Cl_2_ are cancelled by the antiferroelectric arrangements in the crystal structure with a non-polar space group of *Pnma*. If an Li^+^@C_60_ crystal containing polar anions with a polar space group was obtained, the crystal could have a macroscopic electric dipole moment and exhibit ferroelectricity due to a switching of the polar anions and motion of the Li^+^ cations. Ferroelectric crystals of C_60_ encapsulating a polar molecule have been predicted theoretically [32]. Actually, a cubic crystal of C_60_ with a polar water molecule inside (H_2_O@C_60_) shows an increase in the dielectric permittivity according to the Curie–Weiss law at low temperature, although no ferroelectric phase transition is observed down to 8 K [33].

The molecular arrangement of Li^+^@C_60_ molecules in the *bc*-plane shown in figure 2*b* is similar to that of [Li^+^@C_60_](TFPB^−^)·C_4_H_10_O in the *ab*-plane shown in figure 1*b* and the face-centred molecular arrangement in [Li^+^@C_60_](PF_6_^−^). The lattice constants for *b* = 13.89 Å and *c* = 13.96 Å for [Li^+^@C_60_](TFSI^−^)·CH_2_Cl_2_ at 150 K are much smaller than *a* = 17.11 Å and *b* = 20.38 Å for [Li^+^@C_60_](TFPB^−^)·C_4_H_10_O at 260 K, and comparable to the cubic lattice constant of *a* = 14.30 Å for [Li^+^@C_60_](PF_6_^−^) at 150 K. The monolayer lattice in the *bc*-plane of [Li^+^@C_60_](TFSI^−^)·CH_2_Cl_2_ is stacked along the *a*-axis with a shift to the *c*-axis. If the shift is 0, the Li^+^@C_60_ arrangement becomes similar to that in [Li^+^@C_60_](TFPB^−^)·C_4_H_10_O, as shown in figure 1*b*,*c*. If the shift is *c*/2, the Li^+^@C_60_ arrangement becomes similar to the face-centred molecular arrangement in [Li^+^@C_60_](PF_6_^−^). Therefore, the molecular arrangements of [Li^+^@C_60_](TFPB^−^)·C_4_H_10_O (figure 1*b*,*c*) and [Li^+^@C_60_](TFSI^−^)·CH_2_Cl_2_ (figure 2*b*,*c*) can be classified into deformed rock-salt-type structures.

C_60_ cages in [Li^+^@C_60_](TFSI^−^)·CH_2_Cl_2_ also show a severe orientation disorder, as shown in figure 2*d*. The C_60_ cage was modelled by an overlap of three partially occupied C_60_ cages with different orientations. The site occupancies for the three C_60_ orientations are 0.413(3), 0.413(3) and 0.174(5). The two major orientations are equivalent to the crystallographic mirror symmetry. The number of overlapping orientations is less than that in [Li^+^@C_60_](TFPB^−^)·C_4_H_10_O (figure 1*d*). The orientation disorder of C_60_ is strongly affected by the exchange of anion and solvent molecules and is suppressed at low temperature. Anion exchange from larger TFPB^−^ to smaller TFSI^−^ should increase the van der Waals inter-fullerene interactions and electrostatic Li^+^–anion interactions. The measurement temperature of [Li^+^@C_60_](TFSI^−^)·CH_2_Cl_2_ (150 K) is also lower than that of [Li^+^@C_60_](TFPB^−^)·C_4_H_10_O (260 K). These are reasons for the suppression of C_60_ orientation disorder in [Li^+^@C_60_](TFSI^−^)·CH_2_Cl_2_. On the other hand, C_60_ cages in the solvent-free [Li^+^@C_60_](TFPB^−^) show no orientation disorder [21]. It is expected that C_60_ orientations are also ordered in solvent-free [Li^+^@C_60_](TFSI^−^).

The Li^+^ cation in [Li^+^@C_60_](TFSI^−^)·CH_2_Cl_2_ also locates beneath the centre of a hexagon of the C_60_ with the major orientation, as shown in figure 1*e*. The average Li^+^–C distance of 2.42(7) Å is consistent with that in [Li^+^@C_60_](TFPB^−^)·C_4_H_10_O. The Li^+^ position at (0.803, 0.750, 0.040) is close to the gravity centre at (0.763, 0.750, 0.083) for central nitrogen atoms of the six TFSI^−^ anions adjacent to the Li^+^@C_60_ with an interval of 0.98 Å, and, hence, the Li^+^ position is electrostatically consistent with the anion arrangement. The flat thermal ellipsoid for the Li^+^ cation with a site occupancy of 1.0 suggests a large librational motion of the Li^+^ cation. The free rotational motion and positional disorder of the Li^+^ cation in [Li^+^@C_60_](PF_6_^−^) and solvent-free and solvent-containing [Li^+^@C_60_](TFPB^−^) will be suppressed by anion exchange from non-polar PF_6_^−^ and TFPB^−^ anions to polar TFSI^−^ anions.

### 3.3. [Li^+^@C_60_](ClO_4_^−^)

**Figure 3.**
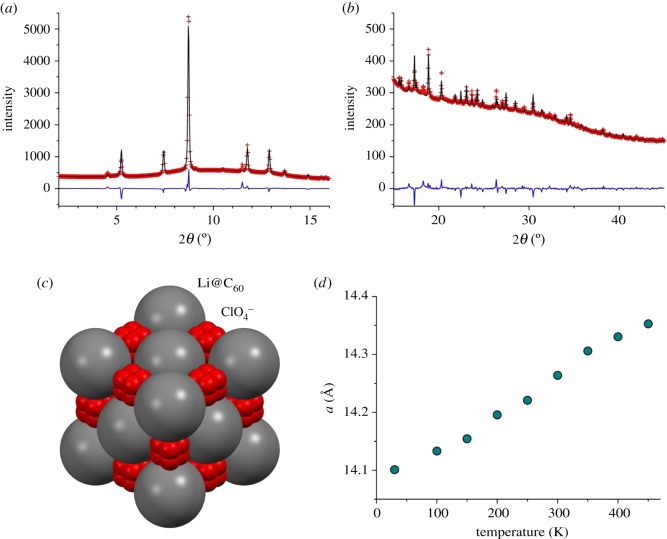
(*a*,*b*) Powder XRD pattern of [Li^+^@C_60_](ClO_4_^−^) at 100 K (X-ray wavelength: 0.649 Å) with a fitting result by the Rietveld refinement. Observed and calculated intensities are plotted by red crosses and black lines, respectively. Deviations between observed and calculated intensities are plotted by blue lines. Low and high 2*θ* angle regions are shown separately in (*a*) and (*b*), respectively. (*c*) Crystal structure model of [Li^+^@C_60_](ClO_4_^−^) viewed along the threefold inversion axis. Grey and red spheres are uniform C_60_ shells and oxygen atoms of disordered ClO_4_^−^ anions, respectively. (*d*) Temperature dependence of the cubic lattice constant of [Li^+^@C_60_](ClO_4_^−^) from 30 to 450 K.

Figure 3*a,b* shows the powder XRD pattern of [Li^+^@C_60_](ClO_4_^−^) at 100 K (X-ray wavelength: 0.649 Å) with a fitting result by the Rietveld refinement. A face-centred-cubic (fcc) structure model similar to the crystal structure of [Li^+^@C_60_](PF_6_^−^) above 370 K was used in the refinement. [Li^+^@C_60_](PF_6_^−^) and pristine C_60_ undergo a phase transition from the fcc structure (space group: $Fm\bar{3}m$) to the low-temperature simple-cubic structure (space group: $Pa\bar{3}$) at 370 K and 260 K, respectively [18,29]. Surprisingly, [Li^+^@C_60_](ClO_4_^−^) has an fcc structure even at 100 K. The cubic lattice constant of [Li^+^@C_60_](ClO_4_^−^) is *a* = 14.13 Å at 100 K, which is smaller than that of [Li^+^@C_60_](PF_6_^−^) at 100 K (*a* = 14.28 Å) and larger than that of pristine C_60_ at 100 K (*a* = 14.06 Å) [18,31]. This relationship is consistent with the fact that a ClO_4_^−^ anion is smaller than a PF_6_^−^ anion.

Figure 3*c* shows the crystal structure model used in the powder pattern fitting. The space group is $Fm\bar{3}m$. The uniform spherical C_60_ shell with a radius of 3.55 Å centred at 0, 0, 0 and the disordered ClO_4_^−^ anion centred at 1/2, 1/2, 1/2 form a rock-salt-type cubic structure. The disordered ClO_4_^−^ anion was modelled by a Cl atom at 1/2, 1/2, 1/2 and partially occupied O atoms at 1/2 ± 0.059, 1/2 ± 0.059, 1/2 ± 0.059 with a site occupancy of 1/2. The disordered structure is given by orthogonal overlap of two ClO_4_^−^ tetrahedra with a Cl–O distance of 1.44 Å. The powder pattern was fitted by using the simple structure model with acceptable reliable factors (*R*_wp_ = 0.036, *R*_I_ = 0.124). However, the reliable Li^+^ position could not be determined due to the multi-site occupation or free rotational motion. The remaining deviations between the observed and calculated intensities in figure 3*a*,*b* are mainly due to non-uniformity of the electron charge densities on the C_60_ shell.

Figure 3*d* shows the temperature dependence of the cubic lattice constant of [Li^+^@C_60_](ClO_4_^−^) from 30 to 450 K. No phase transition was observed in the temperature range. A ternary alkali-doped fulleride Li_2_CsC_60_ also shows no phase transition and has an fcc structure from 50 to 300 K [34]. Disordered occupation of small Li^+^ and large Cs^+^ cations at tetrahedral voids at 1/4, 1/4, 1/4 and octahedral voids at 1/2, 1/2, 1/2 would hinder the orientation ordering of C_60_ at low temperature in Li_2_CsC_60_. By contrast, tetrahedral ClO_4_^−^ anions occupy the octahedral voids in [Li^+^@C_60_](ClO_4_^−^), as shown in figure 3*c*. The mismatch between the molecular symmetry and the site symmetry would hinder the orientation ordering of ClO_4_^−^ and C60 at low temperature in [Li@C_60_](ClO_4_^−^). [Li^+^@C_60_](PF_6_^−^), in which octahedral PF_6_^−^ anions occupy the octahedral voids, shows perfect orientation ordering of C_60_ below 370 K [18].

### 3.4. [Li^+^@C_60_^−^](NiOEP)·CH_2_Cl_2_

**Figure 4.**
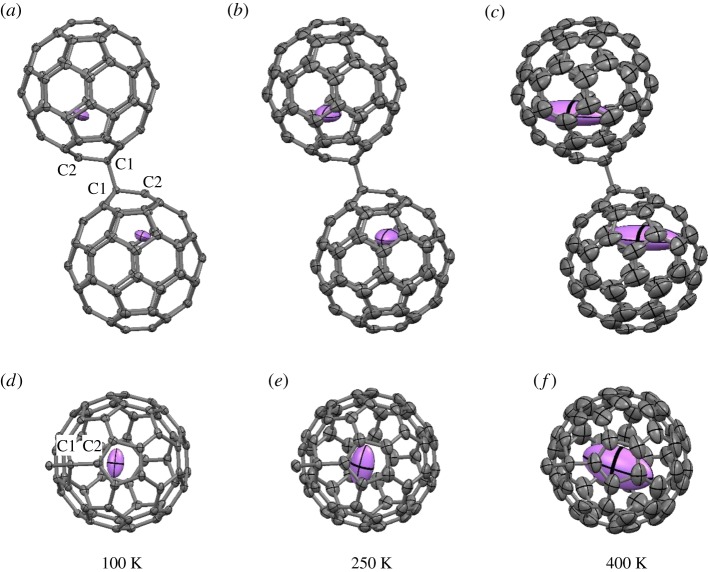
Molecular structure of the (Li^+^@C_60_^−^)^2^ dimer in [Li^+^@C_60_^−^](NiOEP)⋅CH_2_Cl_2_ at (*a*,*d*) 100, (*b*,*e*) 250 and (*c*,*f*) 400 K. (*a*–*c*) View perpendicular to the inter-fullerene single C1–C1 bond. (*d*–*f*) View perpendicular to a pentagon near the violet encapsulated Li^+^ cation. The thermal ellipsoids are drawn at the 50% probability level. Disordered structures at 250 and 400 K and coordinated NiOEP and CH_2_Cl_2_ molecules are omitted.

Figure 4 shows the temperature dependence of the molecular structure of the single-bonded (Li^+^@C_60_^−^)^2^ dimer in [Li^+^@C_60_^−^](NiOEP)⋅CH_2_Cl_2_. The (Li^+^@C_60_^−^)^2^ dimer has a disordered structure by a ratchet-type rotation along the inter-fullerene single C–C bond with a rotation angle of about 39° above the phase transition temperature around 250 K [22]. The disordered structures at 250 and 400 K are omitted in the figure. (C_60_^−^)^2^ dimers in complex crystals of C_60_ with donor and solvent molecules dissociate above 160–220 K [35,36]. By contrast, the (Li^+^@C_60_^−^)^2^ dimers in [Li^+^@C_60_^−^](NiOEP)⋅CH_2_Cl_2_ are thermally stable even at 400 K, as shown in figure 4*c*. It is suggested that the inter-fullerene single C–C bond is stabilized by the encapsulation of Li^+^ cations. Low stability of (C_60_^−^)^2^ dimers is due to the strong repulsion of two negative charges within the dimer. The Li^+^ cation inside the cage which compensates this negative charge contributes essential stabilization of (Li^+^@C_60_^−^)^2^ dimers.

The encapsulated Li^+^ cation is localized with a site occupancy of 1.0 near the carbon atom (C2) nearest to the carbon atom (C1) forming the inter-fullerene single C–C bond (figure 4*a*). The Li^+^–C2 distance is 2.21(1), 2.31(2) and 2.36(6) Å at 100, 250 and 400 K, respectively. The value at 100 K is obviously shorter than the Li^+^–C distance of 2.37(1) Å in [Li^+^@C_60_](PF_6_^−^) at 40 K [19]. It is also noted that the Li^+^ cation in the (Li^+^@C_60_^−^)^2^ dimer locates near the centre of a pentagon involving C2, as shown in figure 4*d,e*, while the Li^+^ cation in [Li^+^@C_60_](PF_6_^−^) locates near the centre of the hexagons [18,19]. The formation of the shorter Li^+^–C bond contributes to stabilization of the (Li^+^@C_60_^−^)^2^ dimer. The inter-fullerene C1–C1 distance shows almost no temperature dependence, which are 1.59(1), 1.59(1) and 1.60(1) Å at 100, 250 and 400 K, respectively.

The thermal ellipsoid of the Li^+^ cation is unusually large perpendicular to the radial direction from the C_60_ centre at 400 K (figure 4*c,f*). The equivalent atomic displacement parameters of the Li^+^ cation and C2 atom are 0.8(1) and 0.077(2) Å^2^, respectively. The thermally induced large librational motion of the Li^+^ cation in the (Li^+^@C_60_^−^)^2^ dimer is basically consistent with the free rotational motion of the Li^+^ cation in [Li^+^@C_60_](PF_6_^−^) above 100 K. The Li^+^ cation bonded to the C2 atom cannot rotate freely in the (Li^+^@C_60_^−^)^2^ dimer even at 400 K.

## 4. Conclusion

We determined structures of cationic Li^+^@C_60_ monomers and neutral (Li^+^@C_60_^−^)^2^ dimers in crystals by SR XRD measurements to understand the electrostatic and thermal properties of the encapsulated Li^+^ cation. [Li^+^@C_60_](TFPB^−^)·C_4_H_10_O and [Li^+^@C_60_](TFSI^−^)·CH_2_Cl_2_ involve severe orientation disorder of the C_60_ cages at low temperature. However, the Li^+^ cations are rather ordered at specific positions by electrostatic interactions from non-polar TFPB^−^ or polar TFSI^−^ anions, which asymmetrically coordinate to the C_60_ cage. [Li^+^@C_60_](ClO_4_^−^) has a rock-salt-type cubic structure similar to the structure of [Li^+^@C_60_](PF_6_^−^). The Li^+^@C_60_ molecules in [Li^+^@C_60_](ClO_4_^−^) are fully disordered with almost uniform spherical shell charge densities by octahedral coordination of ClO_4_^−^ tetrahedra even at 100 K, and show no orientation ordering or structural phase transition unlike [Li^+^@C_60_](PF_6_^−^) and pristine C_60_. Single-bonded (Li^+^@C_60_^−^)^2^ dimers in [Li^+^@C_60_^−^](NiOEP)⋅CH_2_Cl_2_ are stable even at 400 K, while (C_60_^−^)^2^ dimers in a complex of C_60_ dissociate. The formation of Li^+^–C bonds, which are shorter and thermally more stable than Li^+^–C bonds in [Li^+^@C_60_](PF_6_^−^), stabilizes the inter-fullerene single C–C bond and suppresses the free rotational motion of the Li^+^ cation. The variety of structures of Li^+^@C_60_ revealed in this study proves the controllability of the position and motion of the encapsulated Li^+^ cation from outside the C_60_ cage, which would be valuable in future applications of Li^+^@C_60_ as molecular devices. This study shows that the spherical Li^+^@C_60_ cation forms various ionic crystals with common inorganic and organic anions, and the neutral Li^+^@C_60_^−^ consisting of a positive Li^+^ core and a negative C_60_^−^ cage forms thermally stable molecular crystals of covalently bonded (Li^+^@C_60_^−^)^2^ dimers. The results are essential and important to support the strong superatomic character of Li^+^@C_60_.

## Supplementary Material

Crystallographic information file of [Li+@C60](TFPB−)∙C4H10O at 260 K

## Supplementary Material

Crystallographic information file of [Li+@C60](TFSI−)∙CH2Cl2 at 150 K

## Supplementary Material

Crystallographic information file of [Li+@C60−](NiOEP)∙CH2Cl2 at 400 K

## Data accessibility

The CIFs are available as the electronic supplementary material and from the Cambridge Crystallographic Data Centre (CCDC) (www.ccdc.cam.ac.uk/structures) with deposition numbers 1826722, 1826723 and 1826724.

## References

[1] Shinohara H 2000 Endohedral metallofullerenes. Rep. Prog. Phys. 63, 843–892. (10.1088/0034-4885/63/6/201)

[2] Popov AA, Yang S, Dunsch L 2013 Endohedral fullerenes. Chem. Rev. 113, 5989–6113. (10.1021/cr300297r) 23635015

[3] Harneit W 2002 Fullerene-based electron-spin quantum computer. Phys. Rev. A 65, 032322 (10.1103/PhysRevA.65.032322)

[4] Morton JJL, Tyryshkin AM, Ardavan A, Benjamin SC, Porfyrakis K, Lyon SA, Briggs GAD 2006 Bang–bang control of fullerene qubits using ultrafast phase gates. Nat. Phys. 2, 40–43. (10.1038/nphys192)

[5] Mikawa M, Kato H, Okumura M, Narazaki M, Kanazawa Y, Miwa N, Shinohara H 2001 Paramagnetic water-soluble metallofullerenes having the highest relaxivity for MRI contrast agents. Bioconjug. Chem. 12, 510–514. (10.1021/bc000136m) 11459454

[6] Bolskar RD, Benedetto AF, Husebo LO, Price RE, Jackson EF, Wallace S, Wilson LJ, Alford JM 2003 First soluble M@C_60_ derivatives provide enhanced access to metallofullerenes and permit in vivo evaluation of Gd@C_60_[C(COOH)_2_]_10_ as a MRI contrast agent. J. Am. Chem. Soc. 125, 5471–5478. (10.1021/ja0340984) 12720461

[7] Yasutake Y, Shi Z, Okazaki T, Shinohara H, Majima Y 2005 Single molecular orientation switching of an endohedral metallofullerene. Nano Lett. 5, 1057–1060. (10.1021/nl050490z) 15943442

[8] Huang T, Zhao J, Feng M, Popov AA, Yang S, Dunsch L, Petek H 2011 A molecular switch based on current-driven rotation of an encapsulated cluster within a fullerene cage. Nano Lett. 11, 5327–5332. (10.1021/nl2028409) 22081996

[9] Thompson BC, Fréchet JM 2008 Polymer-fullerene composite solar cells. Angew. Chem. Int. Ed. 47, 58–77. (10.1002/anie.200702506) 18041798

[10] Nelson J 2011 Polymer:fullerene bulk heterojunction solar cells. Mater. Today 14, 462–470. (10.1016/S1369-7021(11)70210-3)

[11] Aoyagi S et al. 2010 A layered ionic crystal of polar Li@C_60_ superatoms. Nat. Chem. 2, 678–683. (10.1038/nchem.698) 20651732

[12] Okada H et al. 2012 Preparation of endohedral fullerene containing lithium (Li@C_60_) and isolation as pure hexafluorophosphate salt ([Li^+^@C_60_][PF_6_^−^]). RSC Adv. 2, 10 624–10 631. (10.1039/C2RA21244G)

[13] Matsuo Y, Okada H, Ueno H 2017 Endohedral lithium-containing fullerenes: preparation, derivatization, and application. Singapore: Springer.

[14] Fukuzumi S, Ohkubo K 2013 Long-lived photoinduced charge separation for solar cell applications in supramolecular complexes of multi-metalloporphyrins and fullerenes. Dalton. Trans. 42, 15 846–15 858. (10.1039/C3DT51883C) 24141827

[15] Ohkubo K, Kawashima Y, Sakai H, Hasobe T, Fukuzumi S 2014 Enhanced photoelectrochemical performance of composite photovoltaic cells of Li^+^@C_60_^–^ sulphonated porphyrin supramolecular nanoclusters. Chem. Commun. 49, 4474–4476. (10.1039/C3CC41187G) 23571318

[16] Sakai H, Kamimura T, Tani F, Hasobe T 2015 Supramolecular photovoltaic cells utilizing inclusion complexes composed of Li^+^@C_60_ and cyclic porphyrin dimer. J. Porphyr. Phthalocyanines 19, 242–250. (10.1142/S1088424614501156)

[17] Tran CM, Sakai H, Kawashima Y, Ohkubo K, Fukuzumi S, Murata H 2017 Multi-level non-volatile organic transistor-based memory using lithium-ion-encapsulated fullerene as a charge trapping layer. Org. Electron. 45, 234–239. (10.1016/j.orgel.2017.03.018)

[18] Aoyagi S, Sado Y, Nishibori E, Sawa H, Okada H, Tobita H, Kasama Y, Kitaura R, Shinohara H 2012 Rock-salt-type crystal of thermally contracted C_60_ with encapsulated lithium cation. Angew. Chem. Int. Ed. 51, 3377–3381. (10.1002/anie.201108551) 22374838

[19] Aoyagi S, Tokumitu A, Sugimoto K, Okada H, Hoshino N, Akutagawa T 2016 Tunneling motion and antiferroelectric ordering of lithium cations trapped inside carbon cages. J. Phys. Soc. Jpn. 85, 094605 (10.7566/JPSJ.85.094605)

[20] Suzuki H, Ishida M, Yamashita M, Otani C, Kawachi K, Kasama Y, Kwon E 2016 Rotational dynamics of Li^+^ ions encapsulated in C_60_ cages at low temperatures. Phys. Chem. Chem. Phys. 18, 31 384–31 387. (10.1039/C6CP06949E) 27841436

[21] Okada H, Matsuo Y 2014 Anion exchange of Li^+^@C_60_ salt for improved solubility. Fullerenes Nanotubes Carbon Nanostruct. 22, 262–268. (10.1080/1536383X.2013.812639)

[22] Ueno H et al. 2016 Electrochemical reduction of cationic Li^+^@C_60_ to neutral Li^+^@C_60_^−^: isolation and characterisation of endohedral [60]fulleride. Chem. Sci. 7, 5770–5774. (10.1039/C6SC01209D) 30034715 PMC6022080

[23] Konarev DV, Zorina LV, Khasanov SS, Popov AA, Otsuka A, Yamochi H, Saito G, Lyubovskayaa RN 2016 A crystalline anionic complex of scandium nitride endometallofullerene: experimental observation of single-bonded (Sc_3_N@I_h_−C_80_^−^)^2^ dimers. Chem. Commun. 52, 10 763–10 766. (10.1039/c6cc05550h) PMC5730043 27511304

[24] Voevodin A, Abella L, Castro E, Paley DW, Campos LM, Rodríguez-Fortea A, Poblet JM, Echegoyen L, Roy X 2017 Dimerization of endohedral fullerene in a superatomic crystal. Chem. Eur. J. 23, 13 305–13 308. (10.1002/chem.201703203) 28777885

[25] Sugimoto K, Ohsumi H, Aoyagi S, Nishibori E, Moriyoshi C, Kuroiwa Y, Sawa H, Takata M 2010 Extremely high resolution single crystal diffractometory for orbital resolution using high energy synchrotron radiation at SPring-8. AIP Conf. Proc. 1234, 887–890. (10.1063/1.3463359)

[26] Burla MC, Caliandro R, Carrozzini B, Cascarano GL, Cuocci C, Giacovazzo C, Mallamo M, Mazzone A, Polidori G 2015 Crystal structure determination and refinement via SIR2014. J. Appl. Cryst. 48, 306–309. (10.1107/S1600576715001132)

[27] Sheldrick GM 2015 Crystal structure refinement with SHELXL. Acta Cryst. C 71, 3–8. (10.1107/S2053229614024218) PMC4294323 25567568

[28] Nishibori E, Sunaoshi E, Yoshida A, Aoyagi S, Kato K, Takata M, Sakata M 2007 Accurate structure factors and experimental charge densities from synchrotron X-ray powder diffraction data at SPring-8. Acta. Cryst. A 63, 43–52. (10.1107/S0108767306047210) 17179606

[29] Heiney PA, Fischer JE, McGhie AR, Romanow WJ, Denenstein AM, McCauley JP Jr, Smith AB, Cox DE 1991 Orientational ordering transition in solid C_60_. Phys. Rev. Lett. 66, 2911–2914. (10.1103/PhysRevLett.66.2911) 10043651

[30] David WIF, Ibberson RM, Matthewman JC, Prassides K, Dennis TJS, Hare JP, Kroto HW, Taylor R, Walton DRM 1991 Crystal structure and bonding of ordered C_60_. Nature 353, 147–149. (10.1038/353147a0)

[31] David WIF, Ibberson RM, Dennis TJS, Hare JP, Prassides K 1992 Structural phase transitions in the fullerene C_60_. Euro. Phys. Lett. 18, 219–225. (10.1209/0295-5075/18/3/006)

[32] Cioslowski J, Nanayakkara A 1992 Endohedral fullerites: a new class of ferroelectric materials. Phys. Rev. Lett. 69, 2871–2873. (10.1103/PhysRevLett.69.2871) 10046610

[33] Aoyagi S, Hoshino N, Akutagawa T, Sado Y, Kitaura R, Shinohara H, Sugimoto K, Zhang R, Murata Y 2014 A cubic dipole lattice of water molecules trapped inside carbon cages. Chem. Commun. 50, 524–526. (10.1039/C3CC46683C) 24282827

[34] Hirosawa I, Prassides K, Mizuki J, Tanigaki K, Gevaert M, Lappas A, Cockcroft JK 1994 Orientational disorder of C_60_ in Li_2_CsC_60_. Science 264, 1294–1297. (10.1126/science.264.5163.1294) 17780845

[35] Konarev DV, Khasanov SS, Otsuka A, Saito G 2002 The reversible formation of a single-bonded (C_60_^−^)^2^ dimer in ionic charge transfer complex: Cp*2Cr · C_60_(C_6_H_4_Cl_2_)_2_. The molecular structure of (C_60_^−^)_2_. J. Am. Chem. Soc. 124, 8520–8521. (10.1021/ja0202614) 12121080

[36] Konarev DV, Khasanov SS, Saito G, Otsuka A, Yoshida Y, Lyubovskaya RN 2003 Formation of single-bonded (C_60_^−^)^2^ and (C_70_^−^)^2^ dimers in crystalline ionic complexes of fullerenes. J. Am. Chem. Soc. 125, 10 074–10 083. (10.1021/ja035546a) 12914471

